# Better floors, better health: a theory of change for an improved household flooring intervention in rural communities in Kwale and Bungoma counties, Kenya

**DOI:** 10.1186/s12889-025-21469-1

**Published:** 2025-02-17

**Authors:** Stella Kepha, Hugo Legge, Katherine E. Halliday, Victoria Ochwal, Lynne Elson, Jacinta Mwongeli, William E. Oswald, Beatrice Kakoi, James Wambua, Charles Mwandawiro, Ulrike Fillinger, Rachel Pullan, Doris Njomo

**Affiliations:** 1 Eastern and Southern Africa Centre of International Parasite Control, Kenya Medical Research Institute, Nairobi, Kenya; 2 Department of Disease Control, London School of Hygiene & Tropical Medicine, London, UK; 3 International Centre of Insect Physiology and Ecology, Nairobi, Kenya; 4 KEMRI-Wellcome Trust Research Programme, Kilifi, Kenya; 5 Centre for Tropical Medicine and Global Health, Nuffield Department of Medicine, University of Oxford, Oxford, UK; 6 Jomo Kenyatta University of Agriculture and Technology, Nairobi, Kenya; 7 Global Health Division, International Development Group, RTI International, Research Triangle Park, NC USA

**Keywords:** Housing Flooring, STH, Tungiasis, Enteric, Theory of Change

## Abstract

**Background:**

Household flooring is increasingly being investigated as a determinant of health, however the pathways through which flooring may impact health and wellbeing are not yet well understood. The SABABU study is a cluster-randomised controlled trial evaluating the impact of an improved flooring intervention on soil-transmitted helminthiasis, tungiasis, and enteric infections in Bungoma and Kwale counties, Kenya. This paper presents the findings from a theory of change development process that was undertaken as part of the formative research phase of the SABABU project.

**Methods:**

A co-creation workshop (*n* = 1), stakeholder meetings (*n* = 2), and community meetings (*n* = 2) were held with a range of participants including community members (*n* = 36), village-level leaders (*n* = 28), and local government stakeholders (*n* = 14) to draft and refine the theory of change framework. These meetings were informed by a previous formative research phase conducted in study communities – comprised of household observations, in-depth interviews, and focus group discussions with community members – to investigate daily routines, use of space within homes, and attitudes towards home improvement.

**Results:**

The theory of change framework demonstrates how the improved household flooring intervention aims to reduce prevalence of soil-transmitted helminthiasis, enteric infections and tungiasis and improve psychological wellbeing among children and caregivers. Reductions in infections are predicated on limited contact between improved floors and animals, regular floor cleaning, and household members conducting their daily routines on the new floors. Gains in psychological wellbeing are tied to increased feelings of pride, self-efficacy, and social progress, as well as improved quality of life through reduced morbidity from enteric and parasitic infections.

**Conclusion:**

This study presents a theory of change framework mapping the pathways through which an improved flooring intervention may impact health and wellbeing. The results can be of use to researchers or programmes that are in the design or evaluation phase of a household flooring project in Kenya or other settings where access to improved floors is limited.

**Supplementary Information:**

The online version contains supplementary material available at 10.1186/s12889-025-21469-1.

## Background

Environmental exposures are an important determinant of human health, causing an estimated 23% of global deaths and 22% of global disability adjusted life years (DALYs) in 2017 [1]. Among the different environmental factors driving this burden of disease, housing plays a critical role, impacting infectious disease transmission, non-communicable diseases, and psychological wellbeing [1, 2]. In many regions across the globe, rural communities in particular, continue to grapple with substandard housing conditions which can result in a broad array of negative health outcomes. For example, poor insulation and airflow can increase the risk of asthma, lower respiratory infections (LRIs), heart disease, and lung cancer [3–5], while use of rudimentary construction materials and building practices for walls can increase the risk of exposure to vectors responsible for leishmaniasis, chagas disease, and malaria [6–8]. In addition to these factors there is now increasing attention being paid to household flooring and its relationship with health and wellbeing [9].

The most apparent health-related consequence of flooring is in its capacity to host bacteria, viruses and parasites that can be harmful to human health. Observational studies show that homes with earthen or rudimentary floors (such as untreated wood or bamboo) can sustain a wide range of enteric pathogens as well as skin-parasites, such as the sand flea *Tunga penetrans* [10–15]. Flooring type may also affect other health-relevant environmental and behavioural conditions within a home that can impact the wider wellbeing of occupants. These conditions could include indoor ambient air temperature that can affect sleep quality and psychological wellbeing [16]; the burden of domestic chores, which has been linked to incidence of anxiety and depression among those carrying household responsibilities [17]; and the level of satisfaction that occupants have in their home, which is associated with overall quality-of-life measures [18].

Despite the wide-range of possible health and wellbeing outcomes associated with household flooring, few experimental studies have attempted to evaluate the impact of flooring on health and wellbeing. A 2005 non-randomised evaluation of an improved flooring intervention in urban communities in Mexico found promising reductions in childhood diarrhoea, parasitic infections, and caregiver wellbeing however the pathways through which the intervention achieved these outcomes remains unclear [19]. More recently a feasibility study in Kilifi county, Kenya found that improved floors can be retrofitted into homes and, if done so to a high standard, can yield improvements in domestic hygiene and occupant wellbeing [20].

More evidence is urgently needed to interrogate both the impact of such interventions and the mechanisms through which they yield benefits to health and psychological wellbeing. As household flooring remains a relatively novel intervention the need to fully understand the pathways through which it can impact occupants’ lives is more pronounced. A greater understanding of these pathways will allow for refined intervention design and better insight on contexts in which flooring improvements could be considered as a public health intervention.

Theory of change (ToC) is an approach to programme design and evaluation that seeks to map the ways in which change is expected to occur as a result of an action or intervention. ToC frameworks offer a visual representation of the steps through which change is anticipated to occur in a community or system. ToC frameworks have been cited widely in the environmental health literature as a tool to help design and systematically evaluate interventions [21–24]. While ToC frameworks and other models have been developed to investigate the health consequences of housing, they remain primarily based on data from high-income settings and none have so far referenced the role of flooring [25, 26].

The purpose of this paper is to present the results from a ToC process that was undertaken as part of the formative research and intervention development phase of the Sakafu Bora Afya Bora – Utafiti (SABABU) project (English translation: Better Floors, Better Health - Research). The SABABU project is a three-year study investigating the relationship between household flooring and health and wellbeing in two rural settings in Bungoma and Kwale counties of Kenya. The project builds on the above mentioned study that investigated the feasibility of retrofitting low-cost improved floors in people’s homes in Kilifi county, Kenya [20]. The theory of change presented in this paper was developed through a collaboration between community members, local stakeholders, and members of the SABABU study team. The ToC describes the mechanisms through which it is anticipated that the intervention will achieve expected health and wellbeing outcomes.

## Methods

### Study context

The SABABU project is a cluster-randomised controlled trial (CRCT) study investigating the relationship between household flooring and the health and wellbeing of children and caregivers in two rural settings in Kenya. With regards to physical health, the study is testing the impact of a household flooring intervention on prevalence of soil-transmitted helminthiasis (STH), enteric infections, and tungiasis – an inflammatory skin condition caused by the sand fleas *Tunga penetrans.* The study is comprised of two phases: a formative research component that collected primary data from study communities to inform the design of a household flooring intervention; and the CRCT, intending to evaluate the impact of the flooring intervention on prevalence of soil-transmitted helminthiasis, enteric infections, and tungiasis.

### Study setting

**Fig. 1:**
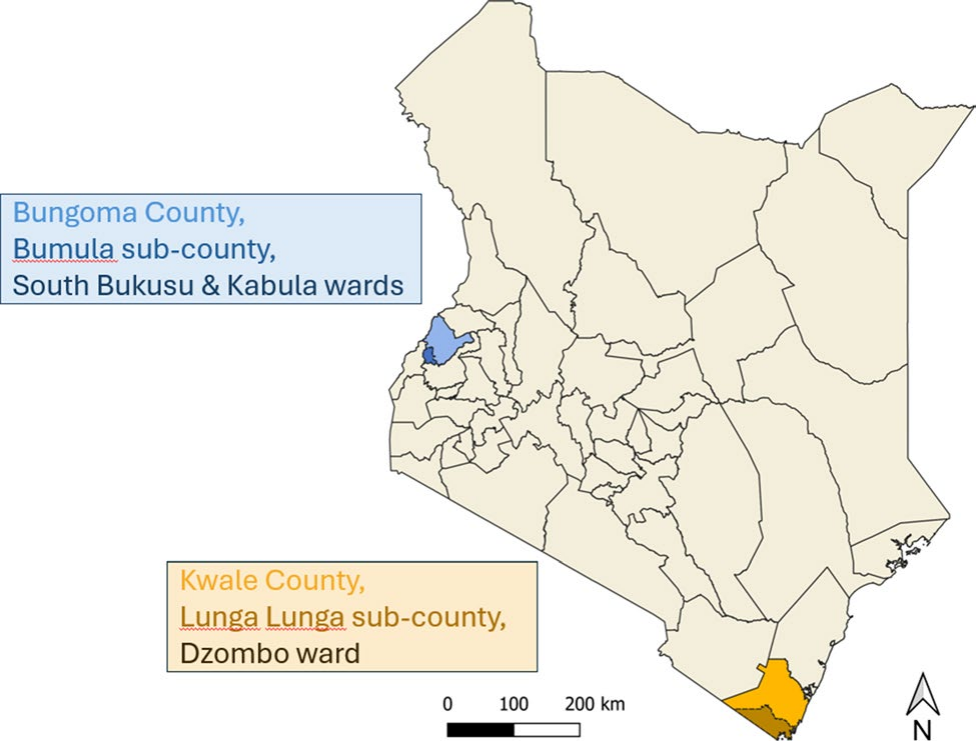
Map of Kenya highlighting sub-counties in which study sites were located

Kwale and Bungoma counties, in Kenya, were selected for the SABABU trial [27]. Formative research was conducted in seven contiguous villages in Dzombo ward, Lunga Lunga sub-county, Kwale and in seven villages across South Bukusu and Kabula wards in Bumula sub-county, Bungoma between the months of May and October 2021. These communities were selected for participation in the study based primarily on known or suspected endemicity for STH and tungiasis and limited presence of improved household floors [28, 29]. The climate in Lunga Lunga is tropical with two distinct rainy seasons (March-May and September-November). An average STH prevalence (predominantly hookworm) of 20% has been recorded and a prevalence of tungiasis upwards of 50% has been reported in some villages [11, 30]. In Bumula, the climate is sub-tropical, and rainfall is experienced throughout the year with a lower prevalence of STH (average of 7.3% any STH) and tungiasis reported as present (Fig. 1).

### Theory of change development process

The ToC development process drew on existing guidance [31] that describes the ToC as a process rather than an output, with a necessity to revaluate frameworks and update them as assumptions are challenged and new understandings emerge. The guidance authors emphasise that ToCs are ultimately guided by individuals’ belief systems, which are in turn influenced by their background and as such it is vital that the process has participation from as wide a group as possible.

Two stages of development were followed to draft a ToC for the SABABU intervention. The first was a formative research phase conducted in study villages and collecting qualitative data around daily routines, use of space within the home, and preferences for household improvements. The second phase involved co-creation workshops and community meetings to synthesise learnings from primary and secondary data and collaboratively develop a ToC conceptual framework.

### Formative research

Formative research was conducted to inform the co-creation workshops and ToC development. The methods and findings of this are presented in detail elsewhere [32], although we describe the methods in brief here for context. The formative work comprised three phases: (1) household observations were conducted in a stratified random sample of eighteen households in each study site to observe daily routines including floor hygiene activities, animal husbandry practices, caregiving, food preparation; (2) caregiver in-depth interviews (IDIs) were undertaken in the same households aimed at exploring motivations underpinning daily activities and triangulating data from the observations; and finally (3) eight focus group discussion (FGDs) per site were held with community members to explore attitudes to building practices, home improvement, and the relative importance of the household floor. Data from observations and IDIs were collated at the household level and reviewed to produce case memos for each activity which, along with the FGD data, were analysed thematically. The findings were reviewed and contrasted between sites and sampling groups to generate insights for the ToC development process.

### Co-creation workshops and stakeholder meetings

A two-day co-creation workshop was held in Kwale County to review results from the formative research and to develop the ToC and a corresponding intervention package. The workshop was attended by stakeholders from the administrative, public health, and housing departments from each study site, as well as representatives from relevant non-governmental organisations that had expertise in housing or other infrastructure-based interventions. In addition, SABABU project collaborators from London School of Hygiene and Tropical Medicine (LSHTM), Kenyan Medical Research Institute (KEMRI), Jomo Kenyatta University of Agriculture and Technology (JKUAT), International Centre for Insect Physiology and Ecology (ICIPE) also participated. By including participants from different locations, sectors, and backgrounds the intention was to ensure a wide range of perspectives were represented during discussions and that assumptions and pre-conceptions would be challenged (Table 1). Results from the formative research activities were first presented to participants, followed by presentations covering ToC theory and processes, as well as approaches to behaviour change. Following these presentations, participants separated into groups and, guided by facilitators, began working on ToC frameworks for each site. Draft frameworks were then presented at plenary for further revisions. Due to similarities in the draft frameworks produced by the individual groups it was agreed that a unified ToC could be developed that incorporated both sites.

Following completion of the co-creation workshop, the SABABU collaborators visited the study sites in both Kwale and Bungoma to present the draft ToC framework and garner feedback. Meetings were first held with sub-county, ward and village-level leaders, including ward administrators, village chiefs, village elders, and Community Health Volunteers (CHVs), before meeting with community members (community members were representatives from the same households that participated in the observations and in-depth interviews). In each meeting the ToC was presented, and critical assumptions were highlighted for particular attention during discussions.

**Table 1:** Theory of change engagement platforms and contributors

Activity	Locality	Sub-county-level participants	Ward-level participants	Village-level participants^	Community member participants~	Project-level participants	Non-governmental participants
Co-creation workshop	Combined*	6	8	0	0	16	5
Stakeholder meetings	Kwale	0	8	0	0	6	0
	Bungoma	2	6	14	0	6	0
Community meetings	Kwale	0	4	14	18	4	0
	Bungoma	0	4	14	18	4	0

*included participants from Kwale and Bungoma sites

^including village elders and community health volunteers

~representatives from the same households that participated in the observations and in-depth interviews

## Results

One two-day combined co-creation workshop and four site-level meetings were held with participants including community members (*n* = 36), village leaders (*n* = 28), local government (*n* = 14), NGOs (*n* = 5), and academic team members (*n* = 16) from the SABABU project. The results from the formative work, which are reported in detail elsewhere [32], were presented at the start of the workshop and the site-level meetings and informed the co-creation process. Please see summary of the key messages taken into the co-creation workshops (Supplementary information 1).

**Fig. 2:**
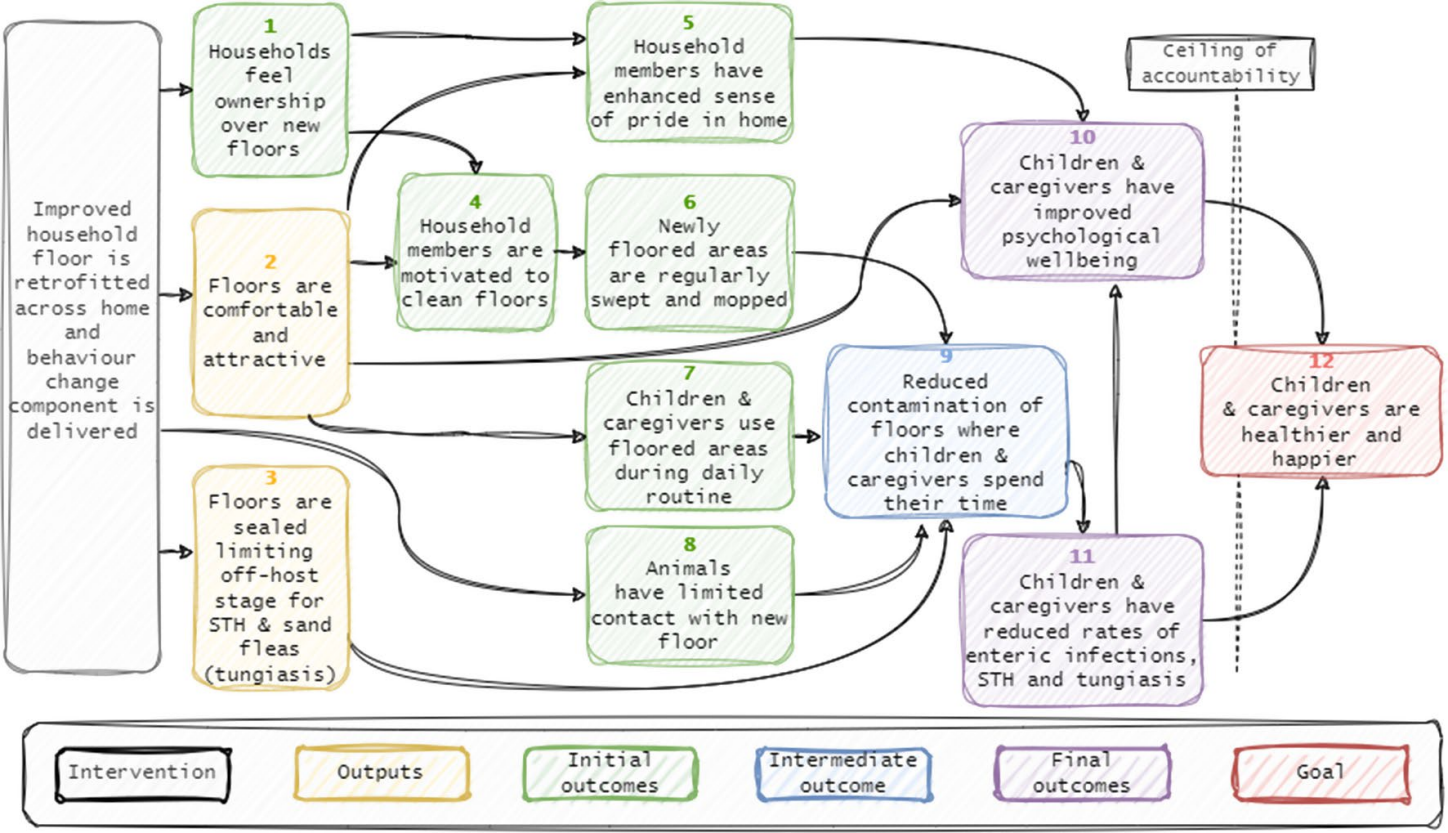
The SABABU theory of change framework

The resulting ToC framework describes how delivery of the SABABU household flooring intervention is expected to reduce prevalence of STH, tungiasis, and enteric infections and improve psychological wellbeing among resident children and caregivers (Fig. 2). Achievement of these outcomes is expected to lead to a longer-term goal of healthier and happier children and caregivers. The different stages of the ToC are organised starting with the intervention activity and then followed by outputs, initial outcomes, intermediate outcomes, final outcomes, and a goal (Table 2).

**Table 2:** Definitions of theory of change stages

Intervention stage	Description
Activity	Action taken by the implementer to deliver the intervention
Output	A direct product of the intervention being delivered
Initial outcome	An initial change occurring within the household as a direct result of the intervention
Intermediate outcome	A subsequent change occurring within the household as a result of initial outcomes being achieved
Final outcome	The ultimate change targeted by the intervention – resulting from initial and intermediate outcomes being achieved
Goal	The long-term outcome towards which the intervention is working. This is not necessarily expected to be achieved within the project timeline.

**Table 3:** Outcomes and their assumptions

ToC item	Outputs and outcomes	Assumptions
11	Children and caregivers have reduced rates of enteric, STH, and tungiasis infections	- Earthen household floors contribute substantially to transmission of enteric and parasitic infections
10	Children and caregivers have improved psychological wellbeing	- Infection rates contribute to health-anxiety among caregivers and reduce quality-of-life for children - Reduced levels of tungiasis in children translates to improved sleep quality, less social stigma, and greater concentration at school - Increased pride in the home translates into overall improvements in psychological wellbeing among caregivers
9	Reduced contamination of floors where children and caregivers spend their time	- Floor hygiene activities are effective at removing pathogens - Animal faeces represents an important pathway through which floors become contaminated with pathogens
8	Animals have limited contact with improved floor areas	- Households are both willing and able to keep animals away from improved floor areas during day and night routines
7	Children and caregivers use floored areas during daily routines	- Intervention floors are comfortable and appropriate for daily activities and time spent on unfloored parts of the home, including the courtyard, is minimal - Children and caregivers spend a substantial portion of their day at the home
6	Improved floor areas are regularly swept and mopped	- Households have requisite materials for sweeping and mopping (e.g. a broom, water) improved floors - Household belongings are kept in raised storage, providing access to improved floor for cleaning - Households have time to regularly sweep and mop improved floors
5	Household members have enhanced sense of pride in their home	- Households feel ownership over improved floor - Household members feel improved floor improves aesthetics of home - Improved floors are associated with social progress by the community - The floor is durable and maintains an attractive appearance over time
4	Household members are motivated to clean floors	- Household members see aesthetic, social, and health-based value in clean floors
3	Improved floors are comfortable and attractive	- Installation of improved floors is done to high standard and meets expectations of household members
2	Improved floors are sealed – limiting off host stage for STH and sand fleas (causative agent for tungiasis)	- Design of improved floor creates a sealed finish that does not crack or crumble over time
1	Households feel ownership over improved floors	- Households contributing to supply of materials, participating in curing of floors, and presence of improved floor within their home translate into feelings of ownership

### Outcome: reduced rates of enteric infections, STH, and tungiasis among children and caregivers

The ToC shows that a reduction in enteric infections, STH, and tungiasis among children and caregivers can be expected by reducing the level of pathogen contamination on household floors where children and caregivers spend their time (ToC item 9). To achieve this state, certain outputs and initial outcomes must be in place; (1) the new floors should create a sealed surface within the home so that the opportunity for the off-host stage for STH species and sand fleas is limited (ToC item 3); (2) animals should have limited or no contact at all with newly floored areas to minimise faecal contamination of floors – a potentially key pathway in the transmission of enteric infections (ToC item 8); (3) newly floored areas should be regularly swept and mopped to reduce pathogen build-up (ToC item 6); and, crucially, (4) children and caregivers must use the newly-floored area as part of their daily routine to maximise exposure to the intervention (ToC item 7).

### Outcome: children and caregivers have improved psychological wellbeing

Caregiver psychological wellbeing is expected to be positively affected for a number of reasons; Participants in the community meetings suggested that the new floors will provide an enhanced sense of pride, which in turn is anticipated to contribute to self-efficacy, and social progress (ToC item 5); the floors are expected to provide greater levels of comfort and convenience for carrying out daily routines (ToC item 2); and the reduction in enteric and parasitic infections among caregivers’ children is expected to reduce health-related anxiety (ToC item 10). Child wellbeing is anticipated to be improved through reduced rates of tungiasis, which is associated with lower quality-of-life scores including shame, disrupted sleep, poor mobility and concentration in school (ToC item 10), reduced STH and enteric infections, and provision of a more comfortable environment within the home for daily activities such as playing and completing school work (ToC item 2).

### The intervention package

**Fig. 3:**
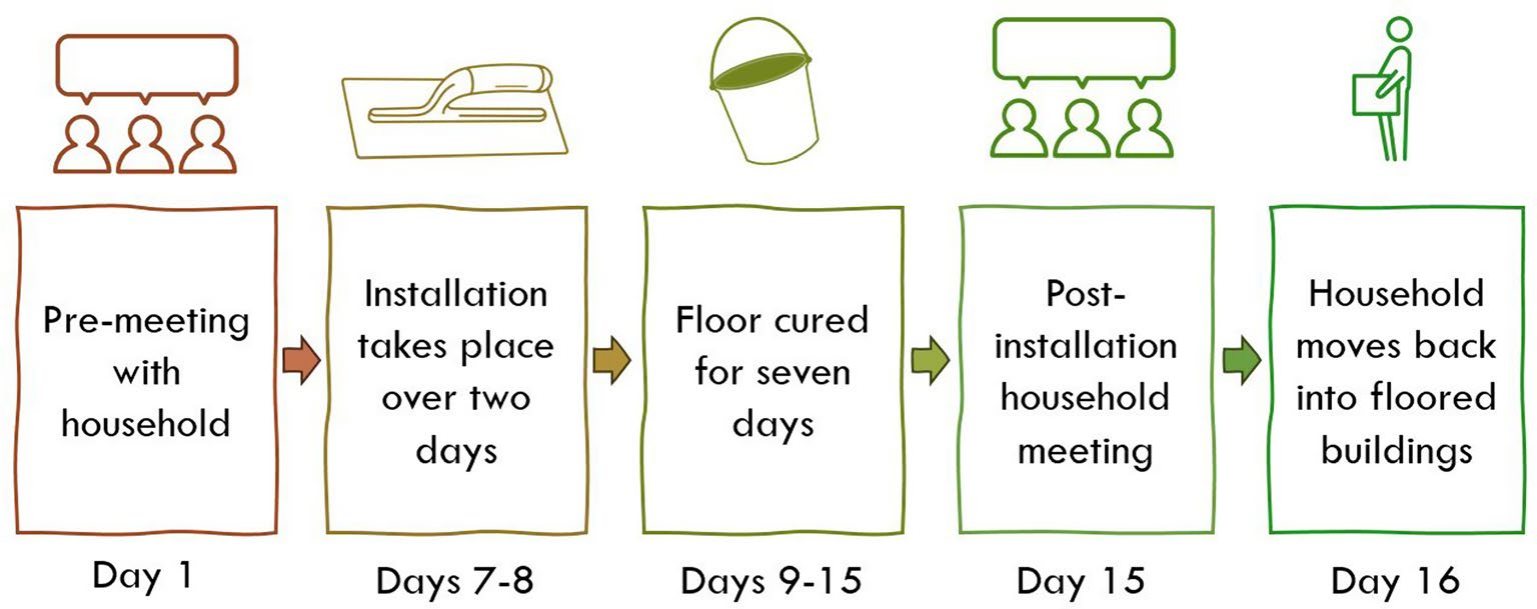
SABABU household flooring intervention cascade

The intervention package will see a sealed, cement-based floor installed across all rooms and buildings within homes, with the exception of dedicated animal sheds and stores, latrines, and buildings that are not structurally secure enough for the new floors to be retrofitted. Installation takes place over a two-day period and is followed by a seven-day curing period during which household members are asked to keep the floor wet by pouring water on it. Following completion of the curing process a post-installation meeting is held with household members to emphasise the behaviours they should follow to maintain and clean their floor effectively (Fig. 3). A locally-adapted A4 laminated infographic that identifies key behaviours is used as a visual aid during this meeting and is left with the household.

Based on the ideas and discussions generated during the co-creation workshops and community and stakeholder meetings the design of the intervention is underpinned by several key concepts:

### Promote ownership of the floor

The success of infrastructure-based interventions are often influenced by the degree to which recipients feel they have ownership over the infrastructure [33, 34]. In the context of the SABABU project, participants in stakeholder and community meetings believed that feelings of ownership over the new floor were crucial to the success of the project. In this sense, the SABABU project benefits from delivering the intervention at the household level, as this contributes towards households’ sense of direct ownership over the new floor. In addition to this, participants at the community meetings indicated that they would be able and willing to source the water required for the installation of the floor and conduct the curing process themselves. These actions have some time and labour costs but they carry no financial burden and were felt to be critical in contributing towards a sense among households that they have been part of the installation process (ToC item 1). The resulting sense of ownership among household members is expected to contribute towards good maintenance of the floor (ToC item 4) – as individuals feel the responsibility to maintain their own property, as well as feelings of pride and social progress (ToC item 5) which we link to the broader wellbeing of caregivers (ToC item 10).

### Maximise exposure to the new floor

For the intervention to be successful in reducing exposure to pathogens, children and caregivers must maximise the amount of time they spend on the newly floored areas. So, the intervention will target all rooms and buildings within homes, with the exception of dedicated animal sheds, latrines, and buildings that may be at risk of collapse if floored. This approach further increases acceptability of the intervention as participants in the community meetings stated that they wanted all rooms in their homes to receive new floors.

### Maintain a hygienic environment

The success of the intervention in reducing prevalence of enteric and parasitic infections in children and caregivers is based on the assumption that the newly floored areas are host to fewer pathogens than the original earthen floors. The cement-based floor is expected to make the environment inherently less hospitable to STH species and sand fleas by limiting the viability of their off-host stages (as these require soil). However, in order to ensure the new floor is kept free from these parasites as well as entero-pathogens, additional steps are required. These include actions that limit the floor’s exposure to contaminants, such as animal faeces, and actions that remove pathogens from the environment, such as sweeping and mopping floors. These actions are promoted through a post-installation meeting with household members and provision of a laminated infographic.

### Assumptions and risks

The ToC framework in Fig. 2 is predicated on a series of assumptions that reflect the cultural, socio-economic, and national backgrounds of its contributors. Articulating these assumptions allows for a clearer understanding of why the intervention may or may not succeed (Table 3).

The core assumption underpinning the success of the intervention’s final outcomes is that household flooring represents a key domain in which the transmission of enteric and parasitic infections is occurring. If the intervention package proves effective in creating a hygienic environment where children and caregivers spend most of their time at home but fails to deliver a reduction in infection prevalence, it will suggest that our assumption is incorrect.

The risks within this ToC are primarily related to the delivery of the intervention, specifically the installation of new floors. If sufficient care is not taken to ensure the structural integrity of buildings before installation takes place then the process could risk damaging the building or causing it to collapse entirely. Further to this, if the installation of a new floor is not completed according to specifications its possible the integrity of the floor could erode over time and cracks and blisters could emerge. In this instance the longevity of the floor could be reduced as well as it becoming harder for households to keep the floors free from contaminants. These risks can be mitigated by only flooring buildings that pass a structural integrity assessment and by ensuring masons adhere to agreed construction guidelines when performing installations of floors.

## Discussion

In this paper, we present a ToC framework that describes the pathways through which an improved flooring intervention is expected to deliver reductions in enteric and parasitic infections while improving psychological wellbeing among children and caregivers in two settings in rural Kenya. The framework identifies the intermediate steps that are required for the intervention to achieve these outcomes and in particular highlights three core themes; (1) the importance of household members having a sense of ownership over the floor; (2) that household members take steps to ensure that the improved floor is kept in a hygienic condition, and; (3) that household members maximise their exposure to the improved floor by carrying out their daily routines on floored parts of the home. To achieve this, the intervention will aim to install new floors in all rooms and buildings within a home, promote ownership of the new floor by involving families in the installation process, and encourage good maintenance and cleaning of the floor by delivering a modest behaviour change component.

While the use of ToC frameworks to help design and evaluate interventions has been widely documented in the environmental health literature [21–24], to our knowledge no previous studies have published a ToC framework for a household flooring intervention. ToCs and other types of conceptual framework investigating the health consequences of housing more broadly have been developed, although remain primarily based on evidence from high-income settings [25, 26]. Of particular note, the ToC framework developed by Thomson et al. in 2015 sought to create an empirically supported comprehensive framework articulating the different components of housing and how they have been shown to impact health [26]. Despite the authors’ attempt to create a comprehensive framework, flooring was not included in the model. This absence reflects the dearth of health-related studies on household flooring and further emphasises the need for research investigating the relationship between household floors and human health.

While flooring has not been explicitly referenced in previous housing-related ToC frameworks, many of the themes referred to in our ToC appear in other frameworks. For example, Thomson et al. assert that increased pride among household members in their home may independently lead to improved mental wellbeing as well as an increased motivation to keep a clean and tidy home [26]. A retrospective ToC developed for the MapSan project in Mozambique identified regular cleaning of sanitation facilities as an important step towards achieving project outcomes [24]. Further to this, the authors suggested that provision of the sanitation infrastructure alone was sufficient to promote regular cleaning of the facilities, and that complementary behaviour change activities likely had little or no-impact on behaviours. This points towards the transformative effect that infrastructure can have on individuals’ routine behaviours when it disrupts their lived-environment.

## Conclusion

Developing a ToC has utility both in the design phase for an intervention – allowing collaborators to think expansively and critically about what steps are necessary for achieving desired outcomes, and in the evaluation phase – providing a blueprint that researchers can use to unpick why a project succeeded or failed to achieve its objectives. In the SABABU study, following a ToC process aided the design of the intervention and the resulting ToC framework will be used to structure the study’s process evaluation, with specific indicators set for each initial and intermediate outcome outlined in the ToC. Beyond the SABABU project, we intend for this ToC to be of use to other researchers, organisations, or programmes that are planning to implement flooring or housing interventions in Kenya and other settings where access to improved floors is limited. Specifically this ToC can act as a starting point for the design of future flooring interventions and their evaluations.

## Electronic supplementary material

Below is the link to the electronic supplementary material.

Supplementary Material 1

## Data Availability

All data presented within this study are contained within the manuscript.
