# Supplementary material for: Better floors, better health: a theory of change for an improved household flooring intervention in rural communities in Kwale and Bungoma counties, Kenya
Source: BMC Public Health. 2025 Feb 17;25:639. doi: 10.1186/s12889-025-21469-1 (PMC11834233; doi:10.1186/s12889-025-21469-1)
Supplement: Supplementary file 1 — Supplementary Material 1 [file 12889_2025_21469_MOESM1_ESM.pdf]

Objectives of the SABABU Formative Research

1) To survey communities and understand their housing and living conditions
2) To look at daily routines and better understand what it is that makes floors dirty
3) To talk to community members and hear their opinions on home improvements and improving floors
4) To create a floor project that meets the needs of community members

Activities of the SABABU Formative Research

Household survey

- Survey all households (between 800-1100 in each site)
- Record housing characteristics
- Record WASH conditions

Household observations and interviews

- Collect detailed housing information
- Observe daily routines
- Conducted in 18 observations per site

Focus group discussions

- Eight discussions in each site
- Discuss attitudes to home improvements and improved floors

Findings of the SABABU Formative Research

Dwelling construction, layout, and suitability for retro-fitting floors

Space	Kwale	Kwale m^2 (range)	Bungoma	Bungoma M^2 (range)
**Kitchen / cooking area**	- Usually the busy room<br>- Usually separate from main building<br>- Acceptability considerations<br>- Practical considerations	7.8 (4.4-13.1)	- Sometimes the busy room<br>- Usually in main building<br>- Practical considerations<br>- Cooking area sometimes outside	11.1 (5.8-21.7)
**Living room**	- High acceptability<br>- Usually in main building<br>- Less busy than kitchen	7.9 (5.3-12.2)	- High acceptability<br>- Sometimes the busy room<br>- Usually in main building	15.6 (5-25)
**Bedrooms**	- Usually does not cover sitting rooms, kitchens and busy spaces<br>- Not busy during the day<br>- Commonly spread across multiple buildings	27.1 (10.6-47.1)	- Some dual-use bedroom/kitchens – these have activities in the day<br>- Dedicated bedrooms not busy during the day<br>- Usually does not include busy room	14.1 (8.4-36.1)
**Whole of main building**	- Likely to include living room and majority of bedrooms<br>- High acceptability<br>- Likely to not include kitchen<br>- Cost	25 (13.1-35.2)	- Likely to include living room, majority of bedrooms, and kitchen<br>- High acceptability<br>- Cost	31.3 (21.7-58.7)
**Whole dwelling**	- Ensures total coverage<br>- High acceptability<br>- Cost	39.8 (14.9-45.4)	- Ensures total coverage<br>- High acceptability<br>- Cost	34.3 (21.7-58.7)

Animal Ownership

- Animal ownership was very high across both sites.
- Animal ownership was between 75% to 99% of households in all villages in both sites.
- Among households that owned animals – cattle, goats, sheep and poultry were most common.
- People tended to own fewer animals in Bungoma compared with Kwale.
- In Kwale and Bungoma – animals spend a lot of time inside dwelling spaces
- Animals gravitate towards wherever there is food
- Children have a lot of direct and indirect contact with chickens, goats, dogs, and cats
- Households that don't own animals still exposed
- Chickens were predominantly kept inside kitchens or other dual-use rooms during the night and then are present in the dwelling during the day
- Goats were often also sleeping in kitchens or dual use rooms. Majority were tied up for grazing during the day, but some cases of free roaming within the dwellings
- Where they are owned, cows tended to sleep in dedicated shelters and spent less time at the dwelling as they are more usually taken out for grazing during the day

Floor cleaning

- In homes with unimproved floors sweeping of courtyards and inside homes is done regularly
- Households cleaned floors as part of their daily routines
- Floor cleaning included both inside buildings as well as courtyards
- Busy spaces gather debris very quickly
- Mostly the responsibility of adult women, but children also participate
- Motivations for floor hygiene included:
    - To make the dwelling look proper or smart
    - To protect against disease; including tics, bedbugs, jiggers and fleas
    - To prevent bad smells in the household

Cooking and food preparation

- In both sites cooking took place inside – either in dedicated kitchens or other rooms
- Rooms where cooking occurred almost always had unimproved floors (even in households with "modern" improved buildings)
- Idea of stove in improved kitchen is a challenge
- Children and caregivers spent a lot of time during the day in kitchens or other places where cooking was occurring

Sleeping arrangements

- Variety of apparatus used for sleeping
- Infants most often sleeping with primary caregivers
- Where a household has multiple rooms for sleeping, children >2 sleep separately from adults
- Little seasonal change in sleeping
